# Establishment and validation of a postoperative predictive model for patients with colorectal mucinous adenocarcinoma

**DOI:** 10.1186/s12957-022-02791-z

**Published:** 2022-10-03

**Authors:** Pengchao Wang, Qingyu Song, Ming Lu, Qingcheng Xia, Zijun Wang, Qinghong Zhao, Xiang Ma

**Affiliations:** Department of General Surgery, The Second Affiliated Hospital of Nanjing Medical University, 121 Jiang jia Yuan Road, Nanjing, 210011 Jiangsu China

**Keywords:** Colorectal mucinous adenocarcinoma (CRMA), Nomogram, Prognosis, Risk stratification, Postoperative

## Abstract

**Background:**

The aim of this study was to develop comprehensive and effective nomograms for predicting overall survival (OS) and cancer-specific survival (CSS) rates in patients with colorectal mucinous adenocarcinoma (CRMA).

**Methods:**

A total of 4711 CRMA patients who underwent radical surgery between 2010 and 2018 from the Surveillance, Epidemiology, and End Results (SEER) database were collected and randomized into development (*n*=3299) and validation (*n*=1412) cohorts at a ratio of 7:3 for model development and validation. OS and CSS nomograms were developed using the prognostic factors from the development cohort after multivariable Cox regression analysis. The performance of the nomograms was evaluated using Harrell’s concordance index (C-index), calibration diagrams, receiver operating characteristic (ROC) curves, and decision curve analysis (DCA).

**Results:**

The study included 4711 patients. Multivariate Cox regression analysis demonstrated that age, tumor size, grade, pT stage, pN stage, M stage, carcinoembryonic antigen, perineural invasion, tumor deposits, regional nodes examined, and chemotherapy were correlated with OS and CSS. Marital status was independently related to OS. In the development and validation cohorts, the C-index of OS was 0.766 and 0.744, respectively, and the C-index of CSS was 0.826 and 0.809, respectively. Calibration curves and ROC curves showed predictive accuracy. DCA showed that the nomograms had excellent potency over the 8th edition of the TNM staging system with higher clinical net benefits. Significant differences in OS and CSS were observed among low-, medium-, and high-risk groups.

**Conclusions:**

Nomograms were developed for the first time to predict personalized 1-, 3-, and 5-year OS and CSS in CRMA postoperative patients. External and internal validation confirmed the excellent discrimination and calibration ability of the nomograms. The nomograms can help clinicians design personalized treatment strategies and assist with clinical decisions.

## Introduction

Colorectal cancer (CRC) ranks third in incidence and second in mortality worldwide [1]. It is estimated that in 2020, in the USA, 104,270 new cases of colon cancer, 45,230 cases of rectal cancer, and approximately 52,980 deaths were caused by CRC [2]. The incidence and mortality of CRC are increasing in China. This may be related to, among other things, the aging of the population, increased westernization of life, retrograde cancer control strategies, and education [3, 4].

There are many pathologies in CRC, of which adenocarcinoma (AC) is the most common. Others include mucinous adenocarcinoma, signet ring cell carcinoma, adenosquamous carcinoma, medusoid adenocarcinoma, and undifferentiated carcinoma, etc. Mucinous adenocarcinoma (MAC) is a unique subtype of adenocarcinoma that accounts for 10–15% of colorectal tumors [5, 6]; however, the incidence is higher in Western countries and lower in Asian countries. According to World Health Organization (WHO) standards, MAC is defined as “>50% of lesions composed of extracellular mucin reservoirs containing malignant epithelium” [7].

In terms of clinicopathological and genetic characteristics, the MAC subtype is characterized by a higher incidence of lymph node infiltration and peritoneal implantation than AC in younger patients, location in the proximal colon, advanced tumors, and a higher proportion of women [8–12]. MAC is characterized by a different molecular pattern that includes microsatellite instability, mismatch repair defects, and overexpression of MUC2 and MUC5AC [9, 13, 14].

Because MAC is more invasive, some researchers suggest more extensive lymph node dissection and a wider resection to avoid the risk of local recurrence [15]. However, at present, the treatment of MAC follows the standard guidelines for CRC, and there are no specific guidelines to standardize the treatment. Targeted surgical resection remains the main treatment mode in CRC, supplemented by preoperative and/or postoperative adjuvant therapy, targeted therapy, and other treatments [16].

Some studies suggest that the prognosis of MAC is worse than that of AC [9–11, 17], although other studies report no significant difference in survival rate and prognosis between MAC and AC [18–20]. The prognosis of MAC remains controversial, underscoring the importance of accurately predicting the prognosis of patients with MAC, which can help clinicians design effective treatment strategies.

The tumor-lymph node-metastasis (TNM) staging system is currently the standard method for predicting the prognosis of patients with CRC and for guiding treatment decisions. However, TNM staging has limitations. It considers a small number of factors and cannot effectively predict the survival of all patients with CRC, particularly in patients with CRMA. In addition, other variables such as age, sex, differentiation grade, carcinoembryonic antigen (CEA) level, perineural infiltration (PNI), tumor deposits (TD), and treatment may also be independent prognostic risk factors in CRC. Therefore, the combination of TNM staging and these variables may be a better predictor of prognosis.

In recent years, the use of nomograms to build prediction models has attracted attention. The nomogram is an intuitive and visual risk prediction graph that quantifies the risk by comprehensively considering and verifying several independent forecasting factors [21, 22]. The nomogram shows a better ability to predict survival than TNM staging [23, 24]. A nomogram for predicting postoperative survival in patients with CRMA has not been developed to date. Here, we developed and validated an intuitive and convenient nomogram for predicting the overall survival (OS) and cancer-specific survival (CSS) of CRMA patients undergoing surgical resection based on the SEER database.

## Materials and methods

### Data sources and patient selection

CRMA patients who underwent radical surgery between 2010 and 2018 were retrieved from the population-based Surveillance, Epidemiology, and End Results (SEER) database and the SEER*Stat program (version 8.4.0, downloaded from http://seer.cancer.gov/seerstat/). The SEER database includes 28% of the American population [25]. Because data are public and open access, no ethics statement or approval was required for the study.

**Fig. 1:**
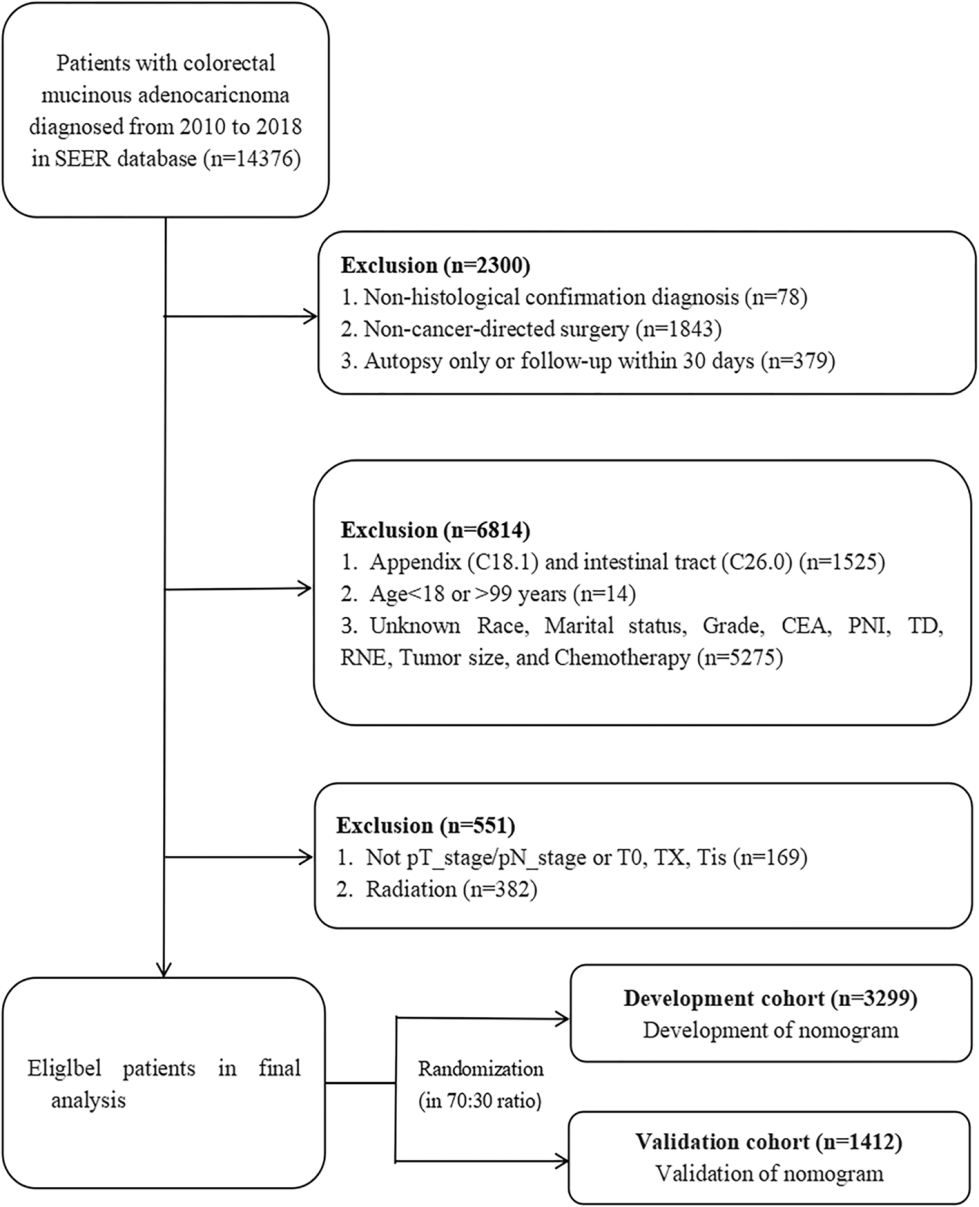
Screening of flow chart for CRMA patients

Inclusion criteria were as follows: (1) diagnosed between 2010 and 2018, (2) mucinous adenocarcinoma (ICD-O-3 Hist/Behav, malignant: 8480/3, 8481/3), (3) CRMA was the only primary tumor, and (4) the use of “site recode ICD-O-3/WHO 2008” data to screen primary tumor location: colon and rectum. Exclusion criteria were as follows: (1) non-histological diagnosis; (2) autopsy only or follow-up within 30 days; (3) no cancer-directed surgery (30-80); (4) tumors in the appendix (C18.1) and intestinal tract (C26.0); (5) age <18 or >99 years; (6) lacking information of race, marital status, CEA, PNI, TD, regional nodes examined (RNE), tumor size, differentiation grade, and chemotherapy; (7) radiation; (8) not pT_stage/pN_stage or T0, TX, and Tis. Although the number of these patients was low, they may skew the survival analysis. Few patients received radiotherapy and were excluded because of concerns that tumor degenerative fragmentation secondary to radiotherapy would be mistaken for TD. A detailed flowchart is provided in Fig. 1. The final study sample comprised 4711 patients. CRMA patients were randomized into development (*n*=3299) and validation (*n*=1412) cohorts in a 7:3 ratio. The development cohort was used to identify prognostic factors and to construct the model, and the validation cohort was used to validate the model internally.

### Variables and endpoints

In this study, 16 variables were extracted including age, race, gender, marital status, tumor site, grade, tumor size, pT stage, pN stage, M stage, CEA, PNI, TD, RNE, and chemotherapy. Patients were divided according to age into two groups, <60 years and ≥60 years. The race was classified as white, black, and others. The tumor size was divided into ≤50 mm and >50 mm. CEA, PI, and TD were classified as negative and positive. Widowed (with domestic partners), single (never married), separated, and divorced cases were classified as unmarried. In accordance with the 7th edition of TNM classification data provided by the SEER database, the tumor stage was reclassified based on the 8th edition of TNM classification. The endpoints included OS and CSS. OS was defined as the time from the diagnosis of CRMA to death from any cause. CSS was defined as the time from the first diagnosis of CRMA to death due to CRMA.

### Construction of nomograms

Cox regression with univariate and multivariate models was applied to evaluate independent risk factors for prognosis. The Kaplan-Meier method and the log-rank test were used to build survival curves. Nomograms were developed based on the independent prognostic risk factors for predicting OS and CSS over 1-, 3-, and 5-year periods. The RMS package for R was used. The scores for each factor in the nomogram of the development cohort were added to determine the total score for each patient.

### Assessment and comparison of model performance

The nomograms were verified by internal (development cohort) and external (validation cohort) validation. The performance of the clinical predictive model was evaluated by discrimination and calibration. The nomogram was verified by model differentiation and model calibration. Model discrimination was evaluated by Harrell’s concordance index (C-index), receiver operating characteristic (ROC) curves, and area under the curve (AUC). The nomogram was compared with the traditional TNM stage by C-index. A higher C-index indicates a higher accuracy of prognosis. The model calibration was used to determine the consistency between the prediction probability of the model and the actual survival rates, which are mainly evaluated by the calibration map. Decision curve analysis (DCA) was performed to compare the reliability of the nomogram, which evaluated whether other diagnostic or prognostic models were superior. The total risk score of each patient in the development cohort was used to determine the optimal cut-off value for dividing CRMA patients. The optimal cut-off value of the total risk score was determined by the x-tile software.

### Statistical analysis

Continuous variables were transformed into classified variables, and classified variables were expressed as quantity and proportion. The correlation between the two cohorts was tested by the *X*^2^ test. Based on a double-tailed statistical test, *P* <0.05 was considered statistically significant. The Kaplan-Meier method was used to build survival curves, and the log-rank test was used for comparisons. In the development cohort, COX regression analyses were used for screening the prognostic indicators. Significant variables in the univariate analysis (*P* < 0.05) were included in the multivariate analysis. All statistical analyses and graphics were performed with SPSS software (version 26.0; Chicago, Illinois, USA) and R software (version 4.1.3).

## Results

### Patient demographics and pathological characteristics

Based on the screening criteria, 4711 CRMA patients who underwent surgery were identified from the SEER database. CRMA patients were randomly divided into two groups: a development cohort (*n* = 3299) and a validation cohort (*n* = 1412) in a 7:3 ratio. Table 1 shows the demographic and pathological characteristics of the patients. There was no significant difference in demographic information and clinical features between the development and validation cohorts. Most patients were ≥60 years old (69.8%), married (54.7%), and white (80.6%). The proportion of women (51.2%) was slightly higher than that of men (48.8%). The right colon (69.1%) was the most common site, followed by the left colon (22.1%), rectum (6.9%), and colon overlap (1.9%). The tumor size >5 cm (57.3%) was the most common. Most tumors were Grade I/II (78%), T3 (59.9%), N0 (49.5%), and M0 (82.3%). Most patients were positive for CEA (50.3), whereas the positivity rates of PNI and TD were 11.8% (negative 88.2%) and 17.5% (negative 82.5%), respectively. Less than half of the patients received chemotherapy (44.3%), and most patients received colectomy with RNE ≥12 (91.9%). The follow-up period of the whole cohort ranged from 1 to 119 months (median, 37 months; average, 44.3 months). The median follow-up period of the development cohort and validation cohort was 38 months and 34 months, respectively.

**Table 1:** CRMA patients’ demographics and pathological characteristics in the development and validation cohort

Characteristics	Total (*n*=4711), *n* (%)	Training group (*n*=3299), *n* (%)	Validation group (*n*=1412), *n* (%)	*P* value
**Age**				
<60	1422 (30.2)	1001 (30.3)	421 (29.8)	0.744
≥60	3289 (69.8)	2298 (69.7)	991 (70.2)	
**Sex**				
Male	2297 (48.8)	1597 (48.4)	700 (49.6)	0.483
Female	2414 (51.2)	1702 (51.6)	712 (50.4)	
**Race**				
Black	505 (10.7)	359 (10.9)	146 (10.3)	0.706
White	3799 (80.6)	2650 (80.3)	1149 (81.4)	
Others (AI, API)	407 (8.6)	290 (8.8)	117 (8.3)	
**Marital.status**				
Unmarried	2135 (45.3)	1527 (46.3)	608 (43.1)	0.045
Married	2576 (54.7)	1772 (53.7)	804 (56.9)	
**Tumor site**				
Left colon^a^	1040 (22.1)	733 (22.2)	307 (21.7)	0.069
Right colon^b^	3257 (69.1)	2299 (69.7)	958 (67.8)	
Overlapping lesion of colon	89 (1.9)	60 (1.8)	29 (2.1)	
Rectum^c^	325 (6.9)	207 (6.3)	118 (8.4)	
**Grade**				
Grade I/II	3674 (78.0)	2568 (77.8)	1106 (78.3)	0.741
Grade III/IV	1037 (22.0)	731 (22.2)	306 (21.7)	
**pT stage**				
pT1	117 (2.5)	93 (2.8)	24 (1.7)	0.073
pT2	504 (10.7)	348 (10.5)	156 (11.0)	
pT3	2821 (59.9)	1989 (60.3)	832 (58.9)	
pT4	1269 (26.9)	869 (26.3)	400 (28.3)	
**pN stage**				
pN0	2332 (49.5)	1652 (50.1)	680 (48.2)	0.177
pN1	1283 (27.2)	904 (27.4)	379 (26.8)	
pN2	1096 (23.3)	743 (22.5)	353 (25.0)	
**M stage**				
M0	3878 (82.3)	2734 (82.9)	1144 (81.0)	0.137
M1	833 (17.7)	565 (17.1)	268 (19.0)	
**Tumor size (mm)**				
≤50	2011 (42.7)	1416 (42.9)	595 (42.1)	0.641
>50	2700 (57.3)	1883 (57.1)	817 (57.9)	
**CEA**				
Negative	2340 (49.7)	1671 (50.7)	669 (47.4)	0.043
Positive	2371 (50.3)	1628 (49.3)	743 (52.6)	
**PNI**				
Negative	4157 (88.2)	2924 (88.6)	1233 (87.3)	0.219
Positive	554 (11.8)	375 (11.4)	179 (12.7)	
**TD**				
Negative	3885 (82.5)	2738 (83.0)	1147 (81.2)	0.157
Positive	826 (17.5)	561 (17.0)	265 (18.8)	
**RNE**				
<12	382 (8.1)	251 (7.6)	131 (9.3)	0.062
≥12	4329 (91.9)	3048 (92.4)	1281 (90.7)	
**Chemotherapy**				
None/unknown	2622 (55.7)	1851 (56.1)	771 (54.6)	0.357
Yes	2089 (44.3)	1448 (43.9)	641 (45.4)	

*CEA *carcinoembryonic antigen, *PNI* perineural invasion, *TD* tumor deposits, *RNE* regional nodes examined

^a^Splenic flexure, descending colon, and sigmoid colon

^b^Cecum, ascending colon, hepatic flexure, and transverse colon

^c^Rectum and rectosigmoid junction

### Cox proportional risk analysis

The prognostic factors correlated with OS and CSS in CRMA postoperative patients were evaluated by univariate and multivariate Cox proportional risk analyses. The results of univariate Cox regression analysis showed that age, marital status, grade, tumor size, pT stage, pN stage, M stage, CEA, PNI, TD, RNE, chemotherapy, and 12 other variables were related to OS (Table 2). In the multivariate Cox regression analysis of OS, 11 independent prognostic factors including age, marital status, grade, pT stage, pN stage, M stage, CEA, PNI, TD, RNE, and chemotherapy were identified (Table 2). Univariate Cox regression analysis showed that 12 variables including age, marital status, grade, tumor size, pT stage, pN stage, M stage, CEA, PNI, TD, RNE, and chemotherapy were significantly correlated with CSS (Table 3). Further multivariate Cox regression analysis indicated that 10 variables including age, grade, pT stage, pN stage, M stage, CEA, PNI, TD, RNE, and chemotherapy were independent factors affecting prognosis (Table 3).

**Table 2:** Univariable and multivariable Cox regression analysis of OS for nomogram in the training cohort

Variable	Univariate analysis		Multivariate analysis	
	HR (95% CI)	*P*	HR (95% CI)	*P*
**Age**				
<60	1		1	
≥60	1.63 (1.44–1.86)	<0.001	1.87 (1.64–2.14)	<0.001
**Sex**				
Male	1			
Female	1.09 (0.97–1.21)	0.134		
**Race**				
Black	1			
White	1.01 (0.85–1.21)	0.881		
Others (AI, API)	0.91 (0.71–1.18)	0.482		
**Martial status**				
Unmarried	1		1	
Married	0.76 (0.68–0.85)	<0.001	0.84 (0.75–0.93)	0.0014
**Primary site**				
Left colon^a^	1		1	
Right colon^b^	1.01 (0.88–1.15)	0.907		
Overlapping lesion of colon	1.33 (0.9–1.97)	0.145		
Rectum^c^	1.1 (0.86–1.41)	0.429		
**Tumor size (mm)**				
≤50	1		1	
>50	1.17 (1.05–1.31)	0.005	1.04 (0.92–1.17)	0.5454
**Grade**				
Grade I/II	1		1	
Grade III/IV	1.8 (1.6–2.03)	<0.001	1.26 (1.12–1.43)	<0.001
**pT stage**				
pT1	1		1	
pT2	1.42 (0.87–2.34)	0.161	1.26 (0.77–2.07)	0.361
pT3	1.98 (1.26–3.13)	0.003	1.31 (0.82–2.08)	0.2591
pT4	4.54 (2.87–7.17)	<0.001	1.93 (1.2–3.1)	0.0068
**pN stage**				
pN0	1		1	
pN1	1.74 (1.52-2)	<0.001	1.5 (1.28–1.76)	<0.001
pN2	3.58 (3.14–4.07)	<0.001	2.42 (2.05–2.85)	<0.001
**M stage**				
M0	1		1	
M1	5.35 (4.75–6.02)	<0.001	3.2 (2.76–3.7)	<0.001
**CEA**				
Negative	1		1	
Positive	2 (1.79–2.23)	<0.001	1.4 (1.24–1.57)	<0.001
**PNI**				
Negative	1			
Positive	2.4 (2.08–2.76)	<0.001	1.21 (1.04–1.41)	0.0146
**TD**				
Negative	1			
Positive	3.23 (2.86–3.65)	<0.001	1.52 (1.31–1.75)	<0.001
**RNE**				
<12	1			
≥12	0.52 (0.44–0.61)	<0.001	0.65 (0.55–0.78)	<0.001
**Chemotherapy**				
No/Unknown	1		1	
Yes	1.29 (1.15–1.43)	<0.001	0.61 (0.54–0.7)	<0.001

*CEA *carcinoembryonic antigen, *PNI* perineural invasion, *TD* tumor deposits, *RNE* regional nodes examined

^a^Splenic flexure, descending colon, and sigmoid colon

^b^Cecum, ascending colon, hepatic flexure, and transverse colon

^c^Rectum and rectosigmoid junction

**Table 3:** Univariable and multivariable Cox regression analysis of CSS for nomogram in the training cohort

Variable	Univariate analysis		Multivariate analysis	
	HR (95% CI)	*P*	HR (95% CI)	*P*
**Age**				
<60	1		1	<0.001
≥60	1.17 (1.01–1.35)	0.036	1.55 (1.34–1.8 )	
**Sex**				
Male	1			
Female	1.05 (0.92–1.19)	0.486		
**Race**				
Black	1			
White	0.86 (0.7–1.05)	0.129		
Others (AI, API)	0.85 (0.64–1.13)	0.269		
**Martial status**				
Unmarried	1		1	0.2546
Married	0.88 (0.77–1)	0.045	0.93 (0.81–1.06)	
**Primary site**				
Left colon^a^	1		1	
Right colon^b^	0.88 (0.75–1.03)	0.103		
Overlapping lesion of colon	1.51 (0.99–2.3)	0.055		
Rectum^c^	1.18 (0.9–1.55)	0.231		
**Tumor size (mm)**				
≤50	1		1	0.1026
>50	1.35 (1.18–1.54)	<0.001	1.12 (0.98–1.29)	
**Grade**				
Grade I/II	1		1	0.0012
Grade III/IV	2.06 (1.79–2.36)	<0.001	1.27 (1.1–1.47)	
**pT stage**				
pT1	1		1	
pT2	1.35 (0.56–3.26)	0.507	1.09 (0.45–2.65)	0.8423
pT3	3.85 (1.72–8.62)	0.001	1.8 (0.8–4.07)	0.1562
pT4	11.64 (5.2–26.05)	<0.001	2.94 (1.29–6.69)	0.0102
**pN stage**				
pN0	1		1	
pN1	3.14 (2.62-3.77)	<0.001	2.1 (1.71–2.58)	<0.001
pN2	7.21 (6.08-8.56)	<0.001	3.5 (2.84–4.32)	<0.001
**M stage**				
M0	1	1		<0.001
M1	8.21 (7.18-9.4)	<0.001	3.58 (3.04–4.22)	
**CEA**				
Negative	1		1	<0.001
Positive	2.55 (2.22-2.93)	<0.001	1.54 (1.33–1.79)	
**PNI**				
Negative	1			0.0398
Positive	2.95 (2.52-3.45)	<0.001	1.19 (1.01–1.41)	
**TD**				
Negative	1			<0.001
Positive	4.52 (3.94-5.18)	<0.001	1.51 (1.29–1.76)	
**RNE**				
<12	1			<0.001
≥12	0.48 (0.39-0.58)	<0.001	0.65 (0.53–0.79)	
**Chemotherapy**				
No/Unknown	1		1	0.001
Yes	2.19 (1.91-2.5)	<0.001	0.77 (0.66–0.9)	

*CEA *carcinoembryonic antigen, *PNI* perineural invasion, *TD* tumor deposits, *RNE* regional nodes examined

^a^Splenic flexure, descending colon, and sigmoid colon

^b^Cecum, ascending colon, hepatic flexure, and transverse colon

^c^Rectum and rectosigmoid junction

### Development of the nomogram

**Fig. 2:**
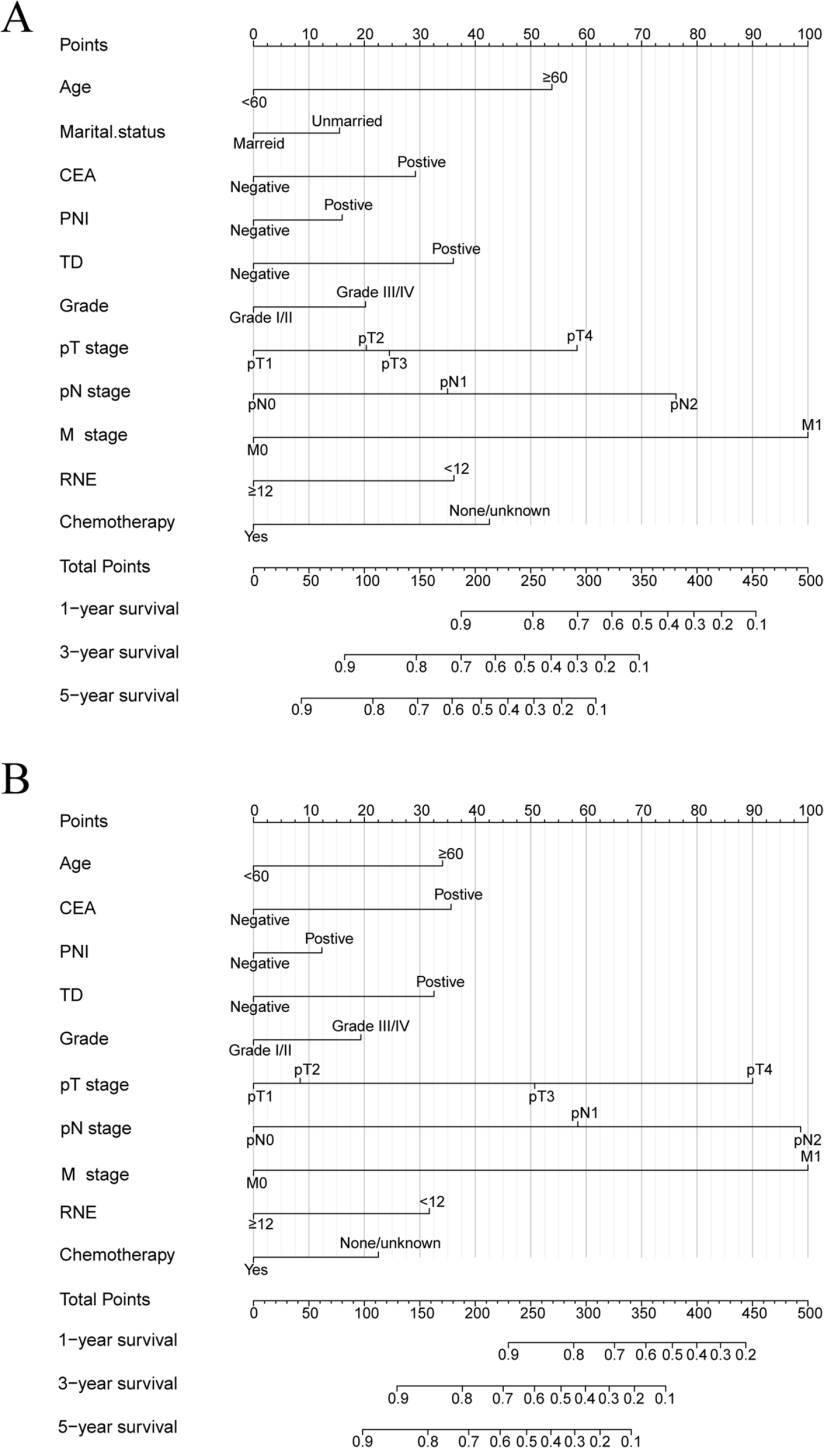
Nomograms predicting 1-, 3-, and 5-year OS (**A**) and CSS (**B**) for CRMA postoperative. The total score of each patient was determined by the sum of the scores of each factor in the nomogram of the development cohort. A vertical line was drawn on the scale below the nomogram according to the total scores, and the corresponding survival rates below the nomogram were the predictive 1-, 3-, and 5-year OS and CSS values

Prognostic variables obtained by multivariate Cox proportional hazard regression were used to develop nomograms for predicting OS and CSS in CRMA postoperative patients in the development cohort. Figure 2 shows the prognostic nomograms for 1-, 3-, and 5-year OS and CSS. The greater the length of each variable in the nomogram, the greater its impact on the survival outcome of patients, and the corresponding scores of each factor are shown in Table 4. The probability of individual survival was determined by adding the scores related to each factor. The detailed scores of prognostic factors in the OS and CSS nomograms are shown in Table 4. For example, using the OS nomogram, patients with stage T3 (25 points), grade III (20 points), CEA positive (29 points), PNI positive (16 points), without chemotherapy (43 points), and older than 60 years (54 points) will have a total of 187 points, which means that the projected 1-year OS is approximately 90%, the projected 3-year OS is approximately 70%, and the predicted 5-year OS is approximately 60%.

**Table 4:** Detailed scores of prognostic factors in the overall survival and cancer-specific survival nomograms

Characteristics	OS nomogram	CSS nomogram
**Age**		
<60	0	0
≥60	54	34
**Martial status**		
Unmarried	16	NA
Married	0	NA
**Grade**		
Grade I/II	0	0
Grade III/IV	20	19
**pT stage**		
pT1	0	0
pT2	20	8
pT3	25	51
pT4	58	90
**pN stage**		
pN0	0	0
pN1	35	58
pN2	76	99
**M stage**		
M0	0	0
M1	100	100
**CEA**		
Negative	0	0
Positive	29	36
**PNI**		
Negative	0	0
Positive	16	12
**TD**		
Negative	0	0
Positive	36	33
**RNE**		
<12	36	32
≥12	0	0
**Chemotherapy**		
No/Unknown	43	23
Yes	0	0

*CEA *carcinoembryonic antigen, *PNI* perineural invasion, *TD* tumor deposits, *RNE* regional nodes examined

### Assessment and verification of nomograms

**Fig. 3:**
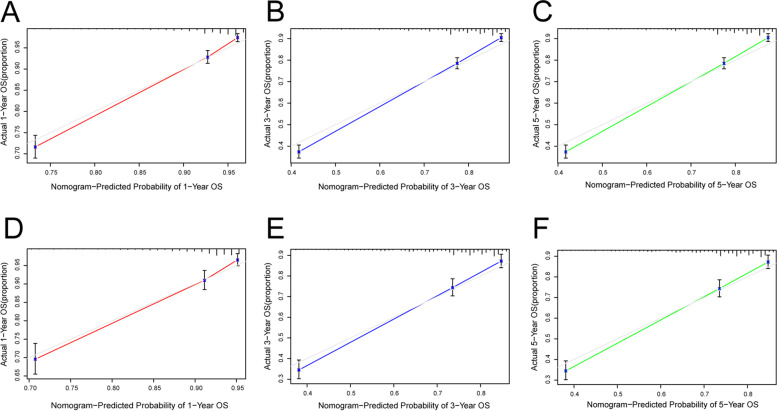
Calibration diagrams for forecasting 1-, 3- and 5-year OS nomograms. **A**–**C** Development cohort. **D–F** Validation cohort

**Fig. 4:**
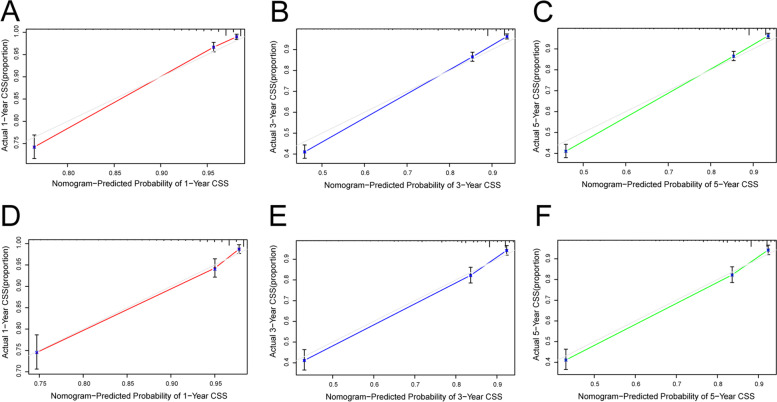
Calibration diagrams for forecasting 1-, 3-, and 5-year CSS nomograms. **A**–**C** Development cohort. **D**–**F** Validation cohort

**Fig. 5:**
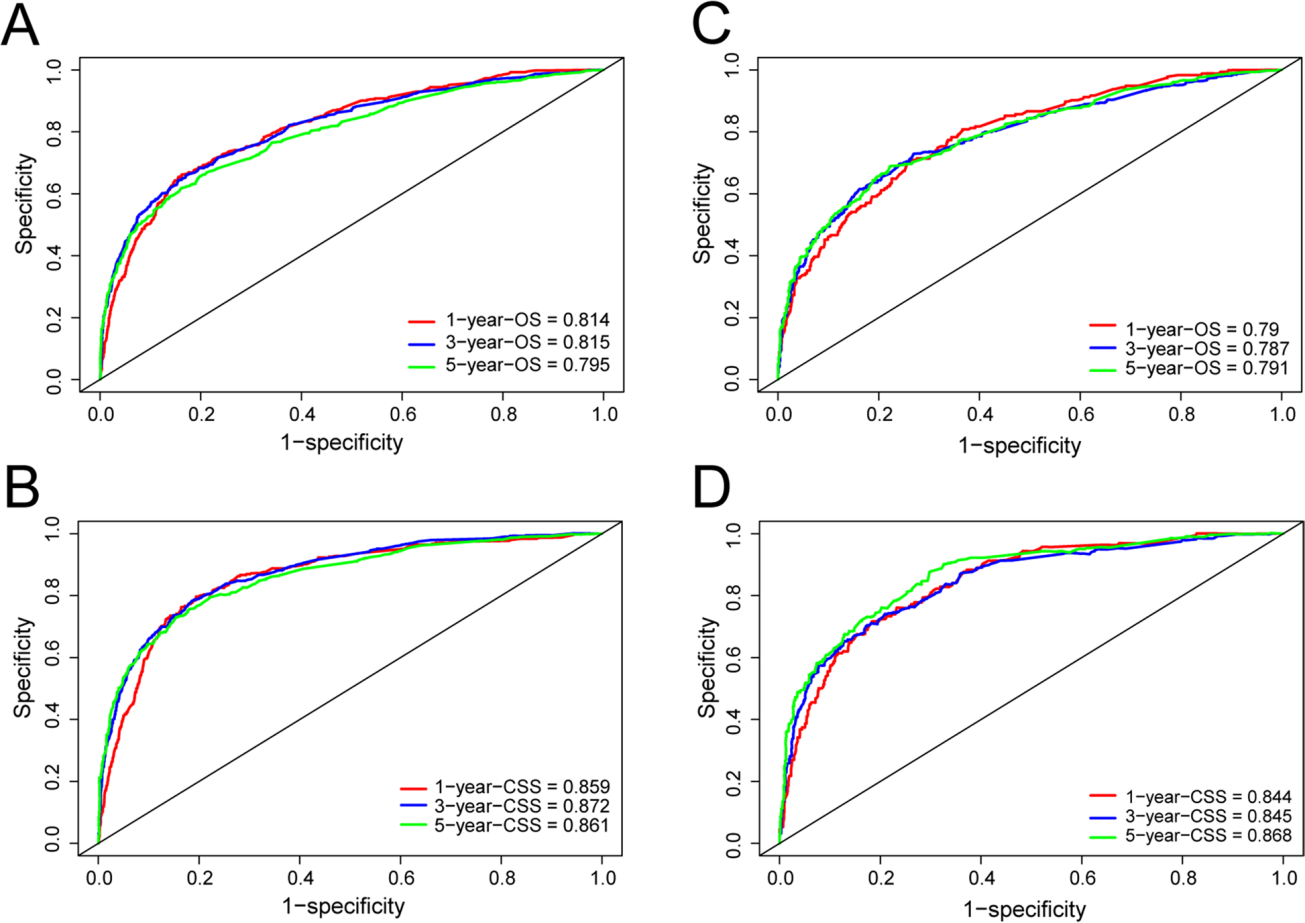
Receiver operating characteristic (ROC) curves predicting OS and CSS. **A**, **B** ROC curves of the development cohort for 1-, 3-, and 5-year OS and CSS. **C**, **D** ROC curves of the validation cohort for 1-, 3-, and 5-year OS and CSS

The C-index of the OS nomogram in the development and validation cohorts was 0.766 [95% confidence interval (CI): 0.753–0.779] and 0.745 (95% CI: 0.724–0.765), respectively, and the C-index of the CSS nomogram was 0.826 (95% CI: 0.813–0.839) and 0.809 (95% CI: 0.788–0.830), respectively. There was no deviation between the calibration curves of the OS (Fig. 3) and CSS (Fig. 4) nomograms in the development and validation cohorts, indicating that the predicted results were in good agreement with the actual observations. Based on the ROC curves and the AUCs of OS for 1, 3, and 5 years, the AUC values of the development cohort were 0.814, 0.815, and 0.795, respectively (Fig. 5), and those in the validation cohort were 0.79, 0.787, and 0.791, respectively. For CSS, the 1-, 3-, and 5-year AUC values of the nomogram were 0.859, 0.872, and 0.861 in the development cohort, respectively, and 0.844, 0.845, and 0.868 in the validation cohort, respectively (Fig. 5). The C-index and AUC values indicated that the nomograms have accurate prediction and sufficient discriminant ability.

### Clinical utility

**Fig. 6:**
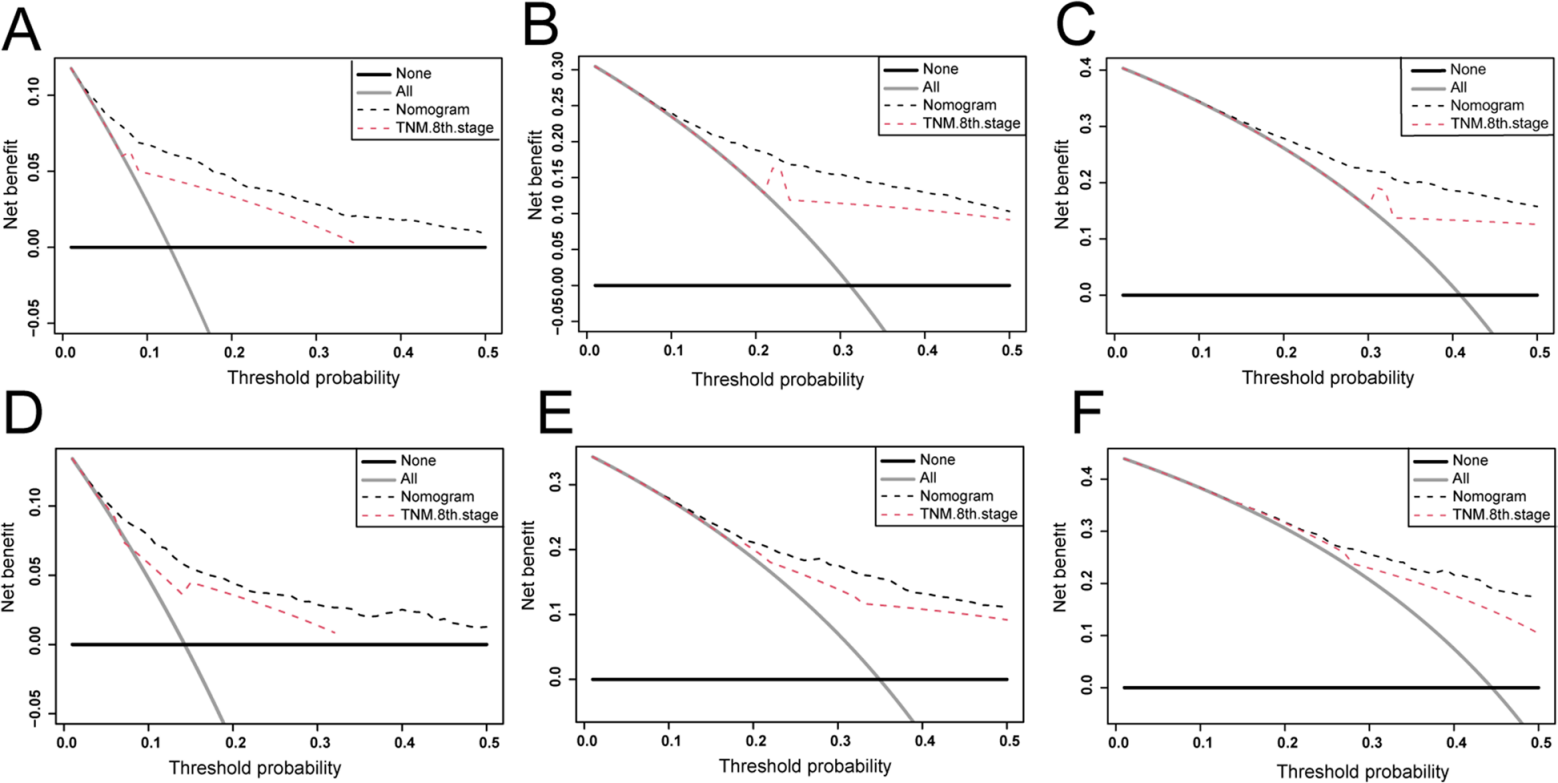
Decision curves analysis (DCA). **A**–**C** DCA curves of the development cohort for 1-, 3-, and 5-year OS. **D**–**F** DCA curves of the validation cohort for 1-, 3-, and 5-year OS

**Fig. 7:**
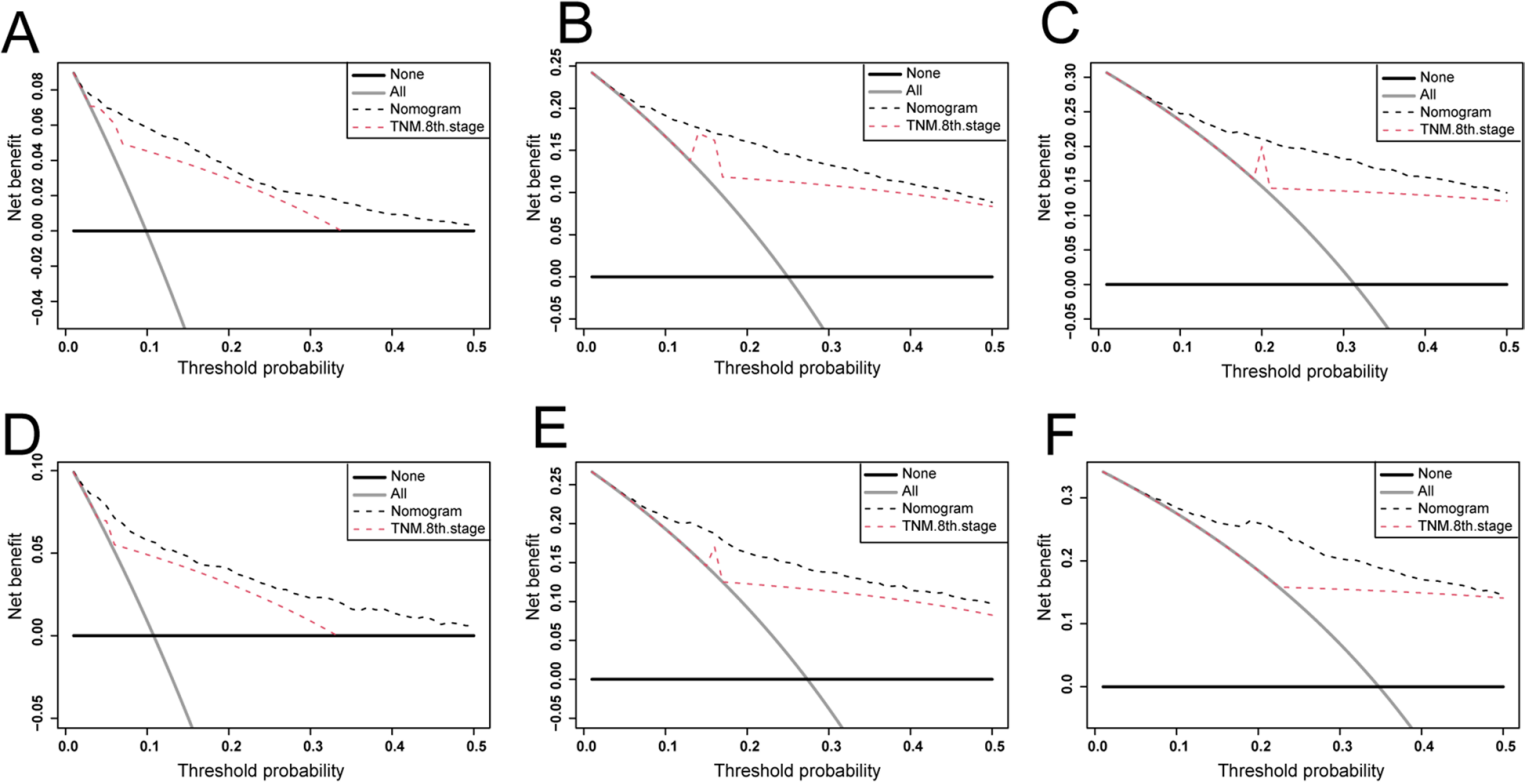
Decision curves analysis (DCA). **A**-**C** DCA curves of the development cohort for 1-, 3-, and 5-year CSS. **D**-**F** DCA curves of the validation cohort for 1-, 3-, and 5-year CSS

DCA is a widely used method to measure clinical utility that is used to analyze the net clinical benefits of predictive models and has several advantages over AUC. In this study, the nomogram and the DCA curve of the 8th edition of TNM staging are shown in Figs. 6 and 7. The DCA of the nomogram had higher net incomes and a wider threshold probability than the 8th edition of TNM staging, indicating that the nomogram had a better clinical utility, which is helpful for clinicians to accurately predict OS and CSS.

### Kaplan–Meier method

**Fig. 8:**
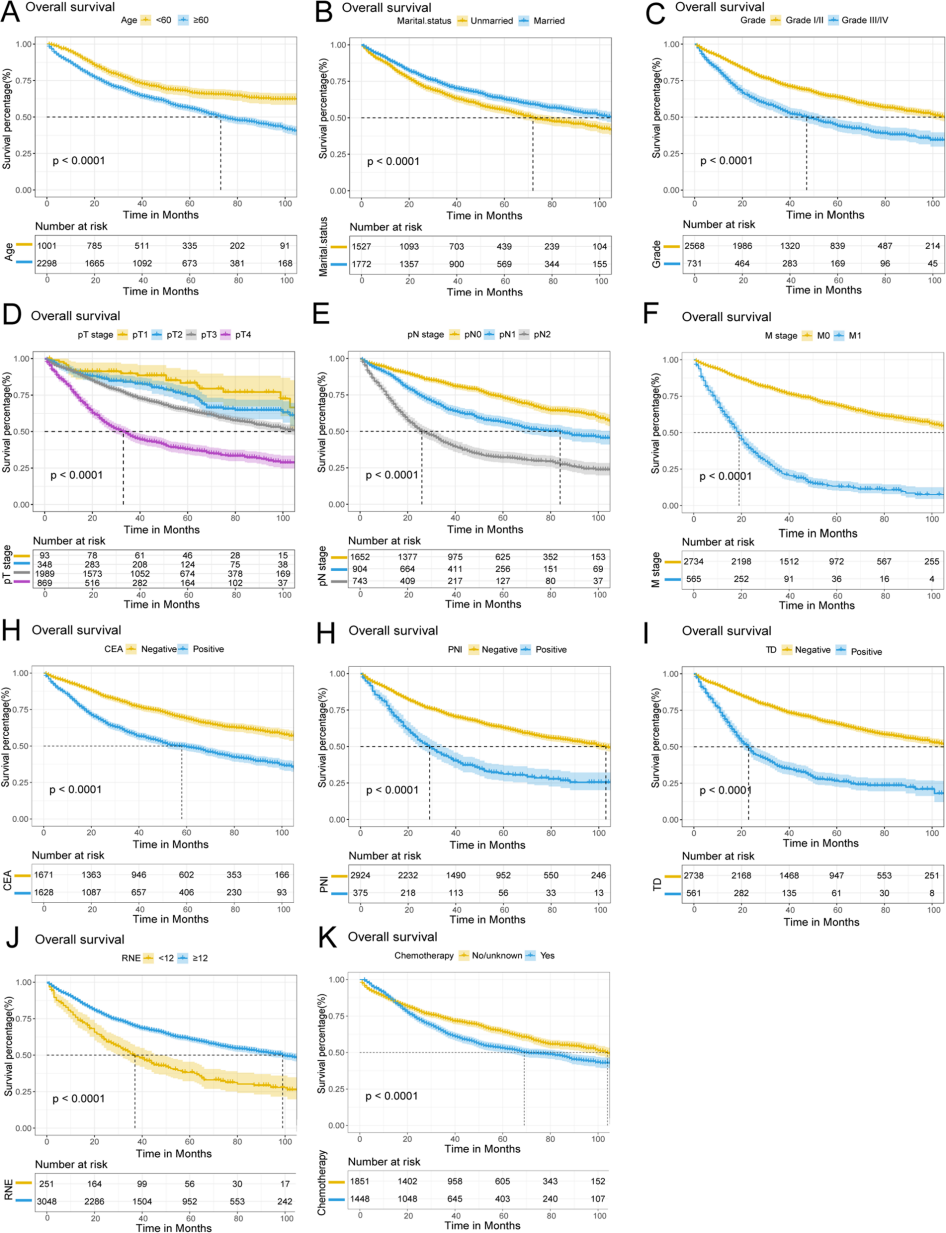
Kaplan-Meier survival curves estimated OS (**A**–**K**) in patients with CRMA. **A** Age, **B** Marital status, **C** Grade, **D** pT stage, **E** pN stage, **F** M stage, **G** CEA, **H** PNI, **I** TD, **J** RNE, and **K** Chemotherapy

**Fig. 9:**
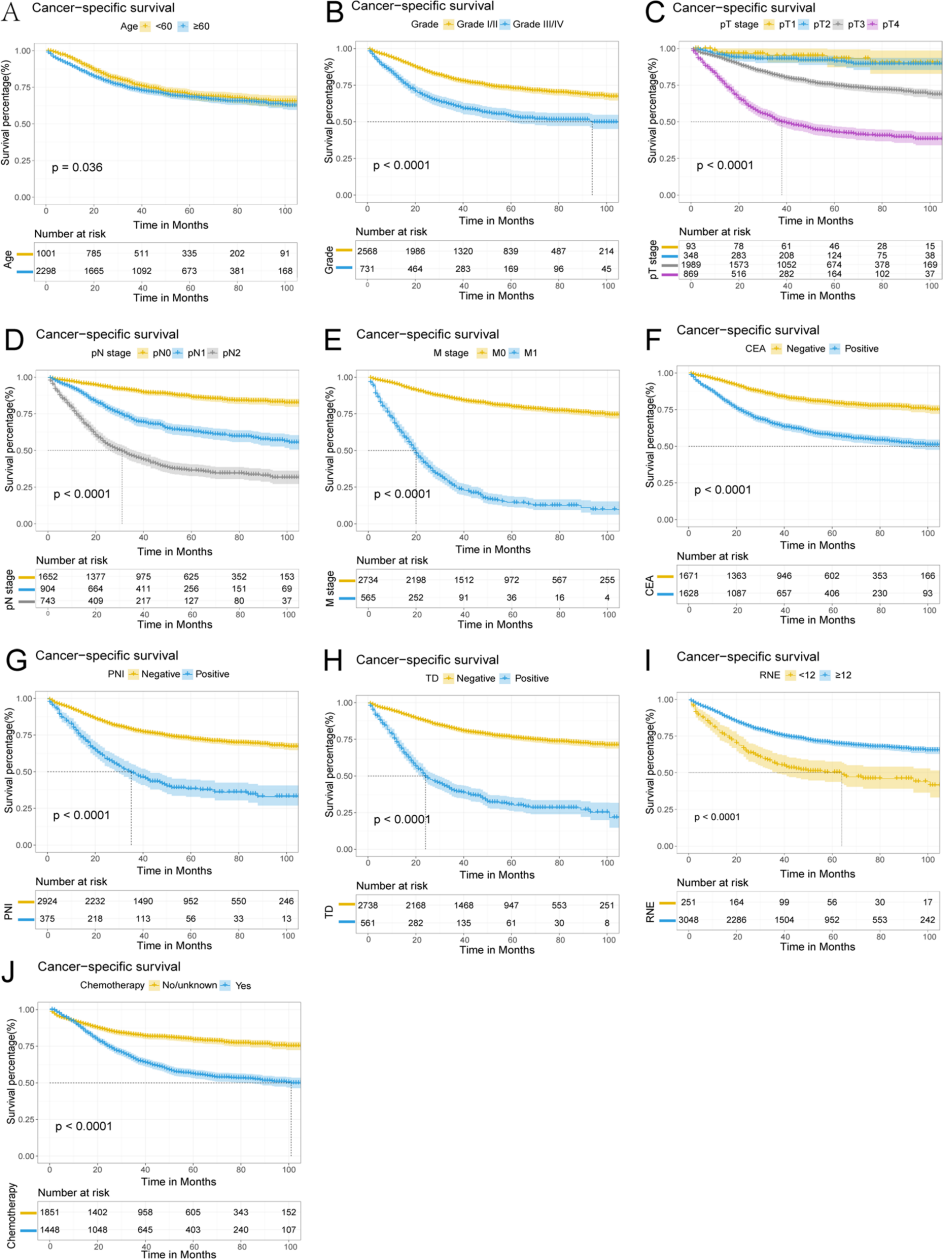
Kaplan-Meier survival curves estimated CSS (**A**–**K**) in patients with CRMA. **A** Age, **B** Grade, **C** pT stage, **D** pN stage, **E** M stage, **F** CEA, **G** PNI, **H** TD, **I** RNE, and **J** Chemotherapy

Kaplan-Meier survival analysis was performed based on significant variables in the multivariate analysis. The results showed that advanced age (≥60 years old), unmarried, III/IV grade, late pT stage, pN stage, and M stage, CEA positive, PNI positive, TD positive, RNE <12, and no chemotherapy were correlated with poor OS (Fig. 8). Advanced age (≥60 years old), III/IV grade, late pT stage, pN stage, and M stage, CEA positive, PNI positive, TD positive, RNE <12, and no chemotherapy were associated with poor CSS (Fig. 9).

### The prognostic nomogram in clinical practice

**Fig. 10:**
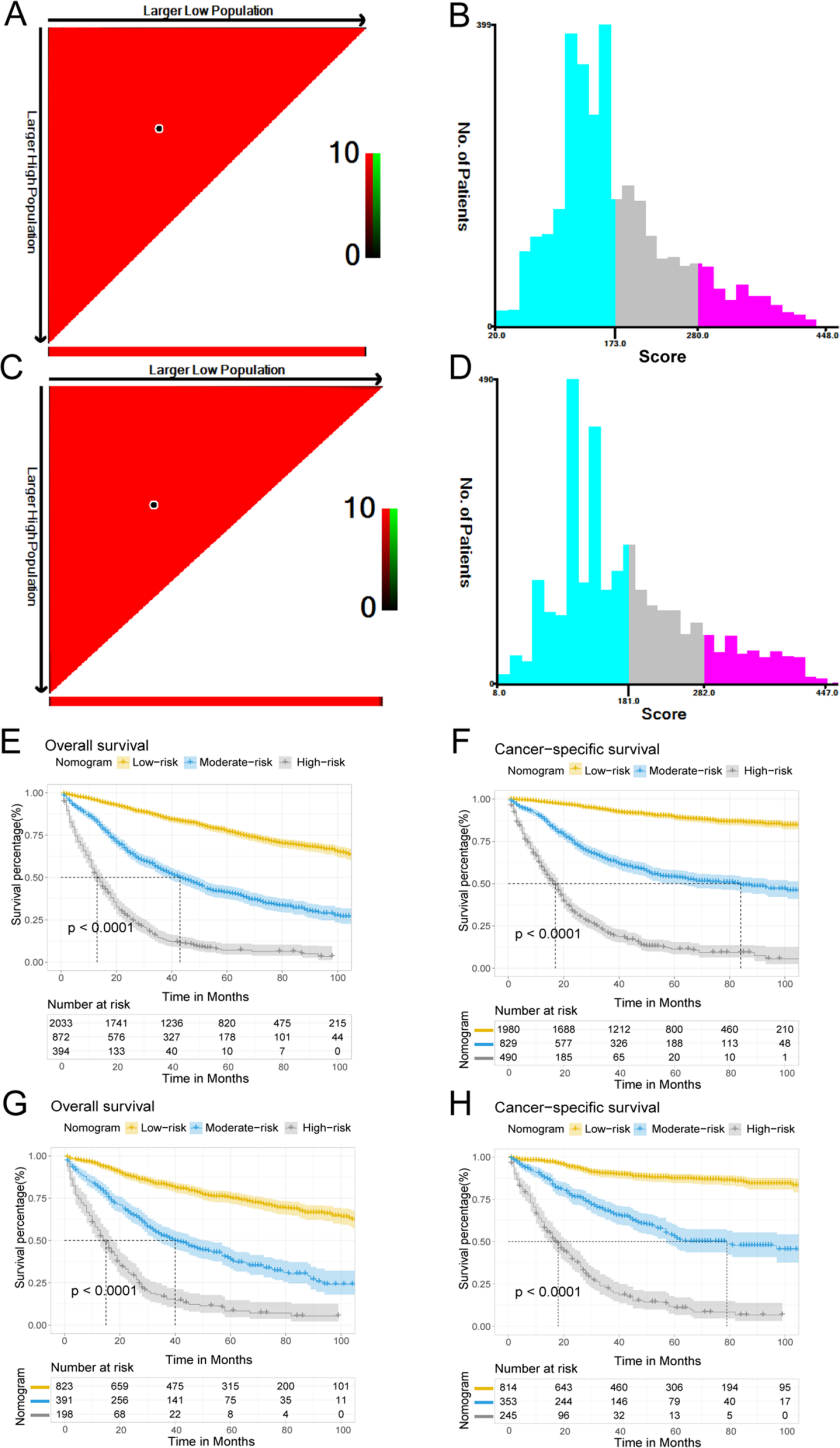
The X-tile software was used to determine the optimal cut-off values of the total risk score of OS (**A**, **B**) and CSS (**C**, **D**) in the development cohort. Kaplan-Meier analysis estimates of the OS and CSS for risk stratification of CRMA patients in the development cohort (**E**, **F**) and the validation cohort (**G**, **H**)

Risk stratification is important to guide patient management. The risk score for each patient was determined by adding the relevant scores for each variable. According to the total risk score, CRMA patients were categorized into low-, medium-, and high-risk groups using X-tile software. The cutoff values for OS were 173 and 281. The cutoff values for CSS were 181 and 283. The OS and CSS of the low-risk group were superior to those of the medium- and high-risk groups (Fig. 10). Similarly, the three risk groups in the validation cohort were categorized according to the same critical values of OS and CSS. Differences in survival rates were also found among the three risk groups in the two comprehensive cohorts, which confirmed that the nomogram had a good ability to predict the prognosis of patients.

## Discussion

The biological behaviors and clinicopathological features of MAC, a pathological type of CRC, remain unclear. MAC differs from AC in many aspects, such as female predominance, late diagnosis, younger age, proximal predominance, lymph node metastasis, and peritoneal implantation. Improving the monitoring, follow-up, and intervention of patients with CRMA can have a major impact on the survival times and prognoses of these patients. Several studies showed that the prognosis of CRMA is worse than that of colorectal adenocarcinoma [9–11, 17]. However, other studies did not report differences in survival or prognosis between CRMA and colorectal adenocarcinoma [18–20]. This suggests that the prognosis of patients with CRMA is heterogeneous. Nomograms have been developed for predicting the prognosis of CRMA patients. Cai et al. developed a nomogram for CRMA, and only patients with stage I–III CRMA were included in the study [26]. Metastasized and non-metastatic CRCs are representations of systemic tumor biology; therefore, excluding patients with metastasis may inherit the limitations of the American Joint Committee on Cancer (AJCC) stages. Previous studies on CRMA were limited to small samples or were combined with signet ring cell carcinoma (SRCC) [18, 20]. SRCC and MAC are two different subtypes, and their prognosis is different, which makes the results unconvincing. Therefore, it is important to perform large cohort studies focused only on CRMA to clarify the prognostic factors and to construct a model for predicting OS and CSS in CRMA postoperative patients.

In this study, 4711 CRMA postoperative patients from the SEER database between 2010 and 2018 were randomly divided into a development cohort (*n* = 3299) and a validation cohort (*n*=1412) at a ratio of 7:3. Prognostic variables were screened using the multivariate Cox proportional hazard regression model. These prognostic factors were used to develop and validate, for the first time, a simple and practical nomogram to assess OS and CSS in postoperative CRMA patients. The nomogram not only combined hematological biomarkers, clinicopathological features, and therapeutic factors, but also included the TNM staging system, which was not done in previous related nomograms. Internal and external validation and comparison with the 8th edition of the TNM staging system indicated that the nomograms have a more accurate predictive ability and prognostic value in CRMA patients, both in the development and validation cohorts. Additionally, the nomograms divided CRMA patients into three risk groups, indicating that the nomograms can be used as a routine tool for predicting the prognosis and survival of CRMA patients.

The results of the study showed that patients aged >60 years have poor survival. The impact of age on the prognosis of CRC was reported previously [27, 28]. We also showed that married patients had a higher survival rate than unmarried patients, which is consistent with previous studies [29, 30]. Studies show that tumor size is not important for the prognosis of CRC [31, 32], which is consistent with the present results. In addition to the conventional TNM staging system, CEA is an important and reliable diagnostic and prognostic marker in CRC, and positive preoperative serum CEA is a stage-independent poor prognostic factor in CRC [33, 34]. The present nomogram showed that CEA-positive patients had a poor prognosis regarding both OS and CSS. The pathology of PNI is characterized by tumors invading nerve structures and spreading along nerve sheaths. Many malignant tumors including CRC show PNI, and increased PNI is associated with a worse prognosis [35, 36]. We found that PNI is associated with several features of advanced CRC such as later tumor stage, larger tumor size, poor differentiation, and distant metastasis. PNI is a common pathological feature of CRC that has a marked influence on prognosis.

Assessment of TD is the focus of an increasing number of studies. The AJCC-8th-TNM classifies any pT stage lesions with negative regional lymph nodes and positive TD as N1c. Nagtegaal et al. found that if TD is classified as N1c lymph nodes and evaluated only in the absence of lymph node metastases, it loses valuable prognostic information [37]. Many studies have confirmed that TD is an independent predictor of poor prognosis in CRC. Liu et al. reviewed two large cohorts including 72,315 patients who underwent radical surgery for CRC and found that TDs played an independent prognostic role by Cox proportional regression analysis [38]. Cohen et al. evaluated 2028 patients with stage III lymph node-positive colon cancer who underwent radical surgery and found that TD positivity was associated with poor survival [39]. In this study, TD-positive patients had a poorer OS and CSS than TD-negative patients. Consistent with previous studies, TD was correlated with a poor prognosis of CRC. The results of this study indicated that tumors with low differentiation/undifferentiation were associated with a poor prognosis. The main treatment for CRC is surgical resection. Studies show that RNE is a significant independent prognostic factor in CRC patients [40, 41]. On the basis of AJCC and National Comprehensive Cancer Network guidelines, pathological specimens after radical surgery for CRC should contain at least 12 regional lymph nodes [42]. In this study, we found that most CRMA patients with RNE ≥12 have better OS and CSS.

The sensitivity of CRMA to chemotherapy remains controversial. Studies show that advanced colorectal MAC is less sensitive to chemotherapy; this may be due to the high rate of microsatellite instability in patients with MAC, which affects their response to chemotherapy [5, 9]. MAC may be insensitive to fluorouracil chemotherapy because of its high expression of topoisomerase-1. Although some studies suggest that MAC is less sensitive to chemotherapy, Hugen et al. proposed that adjuvant chemotherapy is important in the treatment of MAC [5]. The present study showed that chemotherapy is a protective factor for CRMA patients, suggesting that CRMA patients can benefit from chemotherapy. Therefore, chemotherapy is still necessary for patients with CRMA. Notably, the percentage of mucinous components was closely related to prognosis, with higher mucinous composition being associated with worse prognosis. SUMA et al. reported that the percentage of mucus components was significantly associated with tumor aggressive behavior and poor prognosis in colorectal mucinous adenocarcinoma [43]. What is more, Yan et al. reported that the effect of mucin on survival may be associated with its proportion in the lesion, and mucous component >70% may serve as a biomarker for poor prognosis [44]. We found that OS has a lower predictive accuracy than CSS because of the competitive risk. Nonetheless, we opted for the Cox proportional hazard model without competitive risk because the comparison, explanation, and understanding are easier [45].

### Limitations

The present study had several limitations. First, this was a retrospective study, which is associated with inherent bias, and the nomogram needs to be verified by prospective research in the future. Second, some key predictors such as lymphatic infiltration, vascular tumor thrombus, soft tissue metastasis, and microsatellite instability were not extracted from the SEER database. Third, the SEER database lacks detailed treatment information, such as operation modes, radiation doses, chemotherapy, and immunotherapy regimens. In addition, the percentage of mucinous components closely related to the prognosis of MAC patients was also missing, weakening the predictive accuracy and stability of the model. Finally, our research requires additional external data from other sources for validation.

## Conclusion

In summary, we established and validated a postoperative survival prediction model for CRMA patients from the SEER database. Nomograms were more accurate in predicting the survival status of CRMA patients after surgery. The proposed individualized prognostic approach will help clinicians suggest personalized treatment options and assist in risk stratification in patients with CRMA.

## Data Availability

The datasets included in the current study are openly available in the Surveillance, Epidemiology, and End Results (SEER) database at https://seer.cancer.gov/.

## Abbreviations

CRMA: Colorectal mucinous adenocarcinoma
MAC: Mucinous adenocarcinoma
AC: Adenocarcinoma
OS: Overall survival
CSS: Cancer-specific survival
C-index: Concordance index
ROC: Receiver operating characteristic
AUC: Area under the curve
DCA: Decision curves analysis
CEA: Carcinoembryonic antigen
TD: Tumor deposits
RNE: Regional nodes examined
PNI: Perineural invasion
SEER: Surveillance, Epidemiology, and End Results
AJCC: American Joint Committee on Cancer
TNM: Tumor-node-metastasis
